# Molecular basis of resistance to organophosphate insecticides in the New World screw-worm fly

**DOI:** 10.1186/s13071-020-04433-3

**Published:** 2020-11-10

**Authors:** Sophie Tandonnet, Gisele Antoniazzi Cardoso, Pedro Mariano-Martins, Raquel Dietsche Monfardini, Vanessa A. S. Cunha, Renato Assis de Carvalho, Tatiana Teixeira Torres

**Affiliations:** 1 Departamento de Genética e Biologia Evolutiva, Instituto de Biociências, Universidade de São Paulo (USP), São Paulo, SP Brazil; 2 Present Address: Bayer Crop Science, Rua Domingos Jorge, 1100 São Paulo, SP Brasil

**Keywords:** *Cochliomyia hominivorax*, Insecticide resistance, RNA-seq, Condition-specific polymorphisms, Esterase E3

## Abstract

**Background:**

The emergence of insecticide resistance is a fast-paced example of the evolutionary process of natural selection. In this study, we investigated the molecular basis of resistance in the myiasis-causing fly *Cochliomyia hominivorax* (Diptera: Calliphoridae) to dimethyl-organophosphate (OP) insecticides.

**Methods:**

By sequencing the RNA from surviving larvae treated with dimethyl-OP (resistant condition) and non-treated larvae (control condition), we identified genes displaying condition-specific polymorphisms, as well as those differentially expressed.

**Results:**

Both analyses revealed that resistant individuals have altered expression and allele-specific expression of genes involved in proteolysis (specifically serine-endopeptidase), olfactory perception and cuticle metabolism, among others. We also confirmed that resistant individuals carry almost invariably the Trp251Ser mutation in the esterase E3, known to confer OP and Pyrethroid resistance. Interestingly, genes involved in metabolic and detoxifying processes (notably cytochrome P450s) were found under-expressed in resistant individuals. An exception to this were esterases, which were found up-regulated.

**Conclusions:**

These observations suggest that reduced penetration and aversion to dimethyl-OP contaminated food may be important complementary strategies of resistant individuals. The specific genes and processes found are an important starting point for future functional studies. Their role in insecticide resistance merits consideration to better the current pest management strategies.
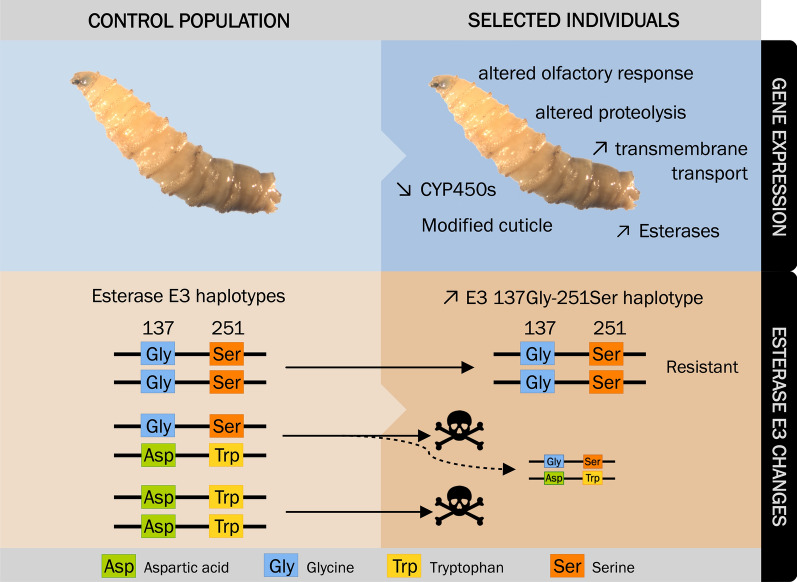

## Background

Pest organisms are defined as those which harm humans and human interests. Detrimental effects include disease, suffering and annoyance, reduction of livestock and agricultural yields, and quality loss of products among others. Pest populations have been almost exclusively controlled by pesticides. The overuse of pesticides, however, represents a strong selective pressure on natural populations leading to the selection of resistant individuals and to the emergence of resistant populations [1, 2]. The replacement of susceptible populations by resistant ones is of worldwide concern as it has greatly reduced the efficacy of pesticides, increasing the damages caused by pests.

The New World screw-worm fly, *Cochliomyia hominivorax*, an ectoparasite of mammals, is particularly troublesome. This species is one of the most important myiasis-causing flies of the neotropical region. Myiasis, an infestation of tissues of vertebrates by dipteran larvae [3], caused by *C. hominivorax* occurs when females oviposit in wounds or exposed tissues of an animal host. Even a small wound (e.g. the bite of a tick) is sufficient to attract a female [4]. When the eggs hatch, larvae promptly feed on the live tissue of the host causing a series of deleterious effects.

*Cochliomyia hominivorax* particularly affects livestock and is responsible for severe economic losses to the cattle industry, estimated at US$336,62 million per year in Brazil alone [5]. One of the main effects is the reduction of leather quality due to the scars caused by the larvae [5, 6], but they also include weight loss, and decrease in milk production. Infestation of calves is life-threatening [7].

Myiases due to *C. hominivorax* are also not rare in humans and generally occur in open wounds [8–10], mucosae [11–15] and particularly in people suffering from poor hygiene or neglect [16]. Infestations in humans are an increasing issue [17] and introductions beyond the New World [18, 19] are a persisting concern.

*Cochliomyia hominivorax* is mainly controlled in Brazil by applying organophosphate (OP)-based insecticides [1]. They are applied directly in the infested wound, targeting larvae feeding off the living tissue of the host. This compound targets the molecule acetylcholinesterase (AChE), which regulates nerve activity by breaking down the neurotransmitter acetylcholine (reviewed in [20]). The inactivation of AChE leads to an overstimulation and blockage of its receptors (e.g. muscle tissue), resulting in paralysis and insect death [20]. Owing to the constant selective pressure from OP application during the last decades, resistant lineages have been strongly selected across several populations of *C. hominivorax* [1, 2].

Although *C. hominivorax* is responsible for great economic loss, few studies have investigated the molecular origin of the resistance phenotype. It is known that mutations at positions 137 and 251 in the enzyme esterase E3, present in *C. hominivorax* [1, 21, 22], enable the breakdown of the OP insecticide preventing the inactivation of AChE [23, 24]. The replacement of Glycine by Aspartic acid at position 137 (Gly137Asp mutation) is involved in diethyl OP (e.g. Diazinon) resistance [24] while the replacement of Tryptophan (Trp) by Serine (Ser) at position 251 (Trp251Ser mutation) confers resistance to dimethyl-OPs (e.g. malathion) [23], and possibly to pyrethroid insecticides [25]. As a first attempt to uncover more candidate genes involved in the dimethyl-OP insecticide resistance, Carvalho et al. [22] sequenced larval and adult (female and male) transcriptomes of *C. hominivorax* and used quantitative PCR of 18 candidate genes coding for metabolic detoxification enzymes to test their association with the resistance phenotype. Of the genes analyzed, only *Cyp6g1* was differentially expressed between control and resistant larvae.

In this study, we also conducted a transcriptome-wide analysis to understand the overall molecular basis of resistance in a *C. hominivorax* population with segregating resistant phenotype by using RNA-seq data. We measured and compared gene expression changes in a subset of individuals of this population selected after a treatment with high concentrations of dimethyl-OP (resistant condition) and non-treated individuals (control condition). We also identified condition-specific polymorphisms, detecting allele frequency shifts caused by the selection. We, therefore, tested the relative contribution of changes in gene expression and polymorphisms in the coding region to the resistant phenotype.

## Methods

### *Cochliomyia hominivorax* colony

In this study, we used a laboratory colony of *C. hominivorax,* which contained OP-resistant individuals segregating the Gly137Asp and Trp251Ser mutations in the esterase E3 gene [1, 21, 22], collected from Caiapônia, Goiás, Brazil on January 2005. The colony was maintained for 11 generations in laboratory conditions according to Carvalho et al. [22]. The same resistant and control samples were used in this study and by Carvalho et al. [22] in their quantitative Polymerase Chain Reaction (qPCR) comparisons. Briefly, for the resistant condition, a sample from the laboratory population was treated with the dimethyl-OP insecticide dichlorvos (2,2-dichlorovinyl dimethyl phosphate; C_4_H_7_Cl_2_O_4_P; Fort Dodge, technical grade) at a lethal concentration (20 mg/L) for 90% of the population (LC90). The insecticide was directly mixed into the diet of the larvae consisting of fresh ground beef supplemented with bovine blood and water (2:1). A total of 500 second-instar larvae were maintained on the insecticide-containing diet for 24 h. Surviving individuals (resistant sample) were collected for total RNA extraction and subsequent analysis. Individuals of the control group were simply sampled from this laboratory population and maintained on the same diet without the insecticide. Individuals from the resistant and the control samples were collected at the same time for RNA extractions.

### DNA and RNA extraction and sequencing

RNA extraction followed the procedure used by Carvalho *et al.* [22]. Total RNA of resistant and control samples was isolated for each individual using TRIzol (Invitrogen^TM^, Thermo Fisher Scientific, Waltham, Massachusetts, USA) from whole bodies of 142 larvae: 44 and 58 surviving individuals respectively from the first and second replicates of the insecticide treatment, and 40 and 60 control larvae from the control group, first and second replicates, respectively. DNase I (Invitrogen^TM^, Thermo Fisher Scientific, Waltham, Massachusetts, USA) was used to remove genomic DNA contamination and mRNA-enriched samples were further purified by using Nucleospin RNA Clean-up columns (Macherey & Nagel, Düren, Germany). Quantification was performed using the fluorometer Qubit Quantitation Platform (Invitrogen^TM^, Thermo Fisher Scientific, Waltham, Massachusetts, USA).

RNA from single individuals of each replicate was pooled for sequencing. The sequencing of the first replicate (single-end reads) was outsourced to the University of Veterinary Medicine, Vienna, Austria and a second biological replicate (paired-end reads) to Laboratório Multiusuários Centralizado, ESALQ-USP (Piracicaba, Brazil). The RNA fragments resulting from the random breaking of the transcripts were converted into a complementary DNA (cDNA) library using the mRNA-Seq Sample Prep Kit (Illumina, San Diego, USA). The samples were sequenced using the HiSeq 2000 (Illumina, San Diego, USA).

For whole genome sequencing of the control population, high-quality DNA was obtained from seven-day-old pupae, as in this stage, no food remains in their gut. DNA extraction was carried out using a phenol/chlorophorm protocol [26]. Samples were prepared with the Illumina TruSeq DNA Low Sample Protocol with the HiSeq SBS v4 High Output Kit (Illumina, San Diego, USA). The library was sequenced in the HiSeq 2500 platform to obtain paired-end (125 bp) DNA sequences. The sequencing service was provided by the Laboratório Multiusuários Centralizado, ESALQ-USP (Piracicaba, Brazil).

### Preprocessing of the reads

To assess the quality of the reads, we used the program FastQC [27], which provided a quick overview of the *C. hominivorax* sequence files. To eliminate the low-quality region of the sequences, we used the program Trimmomatic [28]. This program trimmed the sequences in order to remove read regions with an average quality lower than 15. After trimming, sequences shorter than 20 were discarded. We excluded identical reads to reduce the processing time of the sequences during assembly [29].

### *De novo* transcriptome assembly and annotation

The transcriptome assembly was performed using the program Trinity [30] (version trinityrnaseq_r20140717, --normalize_reads --full_cleanup) in pair-end mode. The single-end reads of the first replicate were inputted as left reads.

The completeness of the assembly was assessed by using BUSCO [31] to search for complete single copy, complete duplicated, and fragmented orthologs within a Diptera database (“diptera_odb9”). The annotation of the transcriptome was carried out using FunctionAnnotator [32] (eukaryotic mode), which uses the NCBI-nr database to find the closest BLAST hits and Blast2GO to assign GO terms.

### Identification of differentially expressed (DE) transcripts

For the differential expression analysis, we removed the redundancy in the transcriptome by clustering the assembled transcripts using CD-HIT-EST [33] (version 4.6, -c 0.95 -M 0 -T 0). This avoided discarding ambiguous read counts (multiple transcript mappings).

To estimate the expression levels of each transcript, we mapped separately the trimmed reads of each condition (Control and Resistant) to the non-redundant transcript set using Bowtie2 [34] (version 2.2.3, ‘--local --very-sensitive-local’ for first single-end replicate and '--local --very-sensitive-local --maxins 1000 --no-mixed --no-discordant' for the second paired-end replicate).

We obtained a table of counts using eXpress [35] (v1.51) and RSEM [36] (v1.2.25) with default parameters. Both programs use an expectation maximization approach and do not require a reference genome for counting. As RSEM does not support gapped alignments, we ran Bowtie2 without the gap argument (“--sensitive --met-stderr --dpad 0 --gbar 99999999 --mp 1,1 --np 1 --score-min L,0,-0.1” and, additionally, “--no-mixed --no-discordant” for the pair-end libraries only).

To test for differential expression between the control and resistant conditions, we used two R packages of the Bioconductor repository; EdgeR [37] (v1.2.25) and DESeq2 [38]. In both, counts were normalized by the library size. We considered for further examination the transcripts for which the false discovery rate (FDR) was below 0.05 and the absolute fold change higher than 2 in all analyses performed (eXpress or RSEM counting with either DESeq2 or EdgeR testing).

### Variant analysis

To test if the resistant phenotype could be a result of Single Nucleotide Polymorphisms (SNPs) in the transcripts, we conducted a variant analysis using the pipeline KisSplice [39] (version 2.4.0). This tool was designed to identify and test for differential allelic usage in RNA-seq data without the need of a reference genome. Briefly, kisSplice locally assembles the regions which surround variants (kissplice -s 1). The localisation of the polymorphic regions is then determined by aligning them to the transcriptome using BLAT [40] (version 34, -minIdentity=80). The R package KissDE was then used to identify which variants were enriched in one condition *versus* the other. Finally, we filtered the polymorphisms predicted to have a functional impact (non-synonymous SNPs within the coding region) by using KisSplice2reftranscriptome (version 1.2.2), which uses the longest open reading frames (ORFs) identified by Transdecoder [41] (version 3.0.1). For further analyses, we considered the condition specific, non-synonymous SNPs displaying (i) a magnitude of differential allelic expression of at least 20%, (ii) a coverage of at least 20 per replicate and (iii) an adjusted P-value below 0.01.

Because we found transcripts expressing predominantly one allele in the control condition (allele contributing to more than 90% of the total expression of that transcript), but expressing two alleles in the resistant condition, we used KisSplice to find SNPs in the genomic reads from the control condition, following the steps described above. From this result, we could determine if both alleles were indeed present in the control population.

Specifically for the esterase E3 (transcript c13624_g1_i1), we analysed the positions 137 and 251, which correspond to the respective positions of the two point mutations (Gly137Asp and Trp251Ser) of resistant individuals of *C. hominivorax*. For this, we counted the polymorphisms at those positions and compared their frequencies in each condition. We set a 1% cut-off to remove sequencing errors.

### Gene Ontology (GO) analysis

We conducted a GO enrichment analysis to identify the GO terms over-represented in the gene sets impacted by the condition (either displaying a differential expression or containing condition-specific SNPs) *versus* those not impacted. This analysis was performed with the Fisher’s exact test, implemented in the program Blast2GO [42] (version 5.2.5), using a False Discovery Rate (FDR) threshold of 0.05. We tested separately the up- and down-regulated gene sets. The genes containing condition-specific SNPs were analysed together. The lists of over-represented GO terms were reduced to the most specific terms [42](version 5.2.5).

## Results

### Transcriptome assembly and annotation

Transcriptome assembly was performed on trimmed and collapsed reads (Additional file 1: Table S1). Collapsing of identical reads significantly reduced computation time to build the transcriptome. The Trinity-assembled transcripts were clustered to reduce redundancy (see Methods). The final reduced transcriptome consisted of 33,038 transcripts (average length of 809.75bp, SD 1001.32 and N50 1493) of which 11,055 (33.5%) were assigned at least one GO term and 16,541 (50.0%) were annotated with a blast hit to the NCBI-nr database. The blast hits belonged mostly to *Lucilia cuprina* (12,055, 72.9%), *Musca domestica* (1411, 8.5%) and *Stomoxys calcitrans* (1209, 7.3%), which is concordant with the divergence times between *C. hominivorax* and each of these species [43].

### Expression profiling comparison

**Fig. 1:**
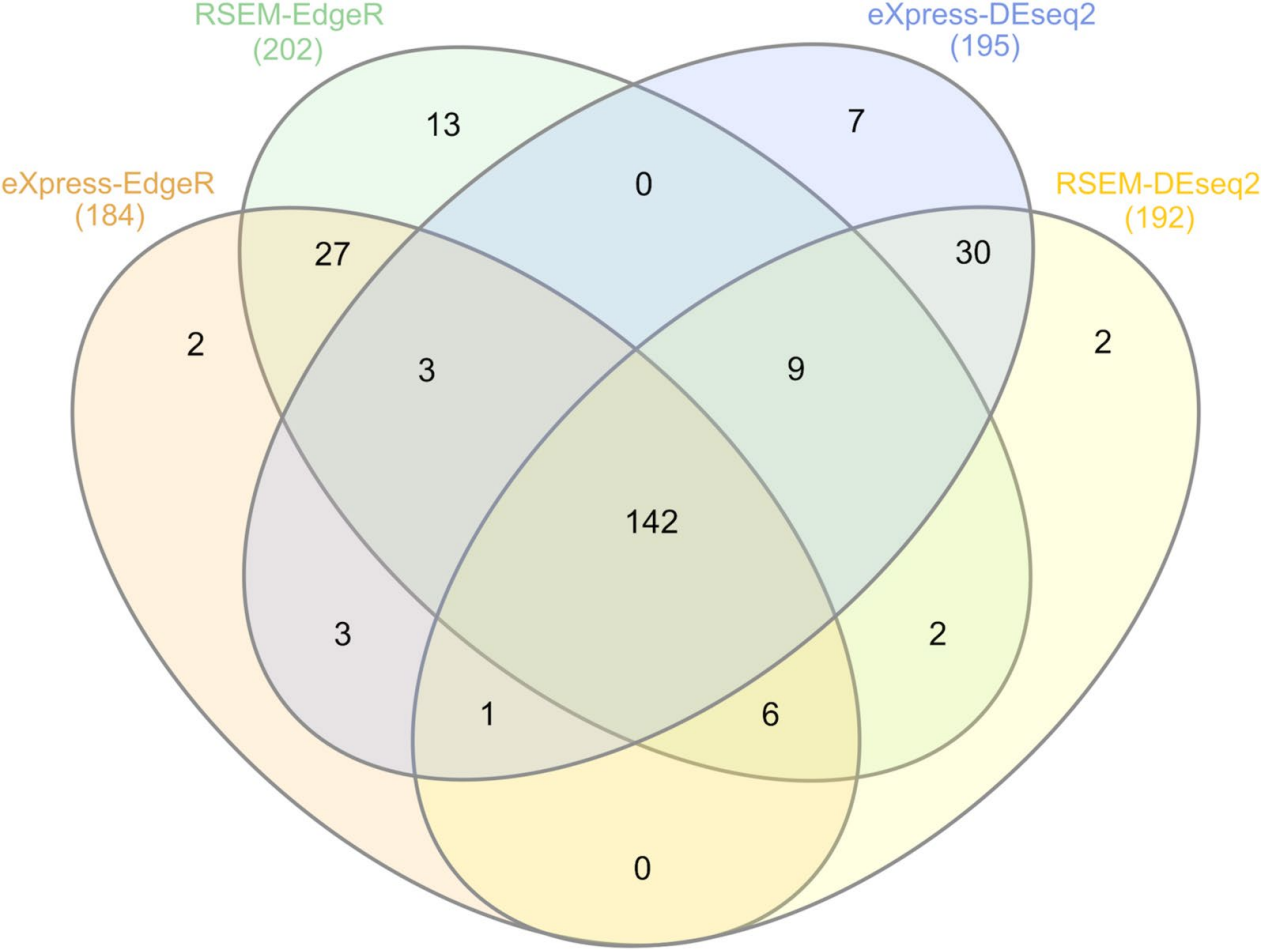
DE transcripts found by DEseq2 and EdgeR on counts generated by eXpress and RSEM. This diagram shows the overlapping results among four different strategies to identify and validate DE transcripts (control *versus* resistant). Most of the DE transcripts were found in all comparisons (142 transcripts)

To identify the differentially expressed (DE) transcripts between the control and the resistant conditions, we used two methods to calculate the abundance of each transcript (eXpress and RSEM) as well as two R packages to test the differential expression (DEseq2 and EdgeR, see Methods). All combinations of counting and testing methods resulted in a similar set of DE transcripts (Fig. 1). Only the common DE transcripts (142) found by all analyses were further examined. Of these, 77 were found up-regulated and 65 down-regulated in the resistant condition compared to the control condition.

#### Response to stress and immune-related transcripts

**Fig. 2.**
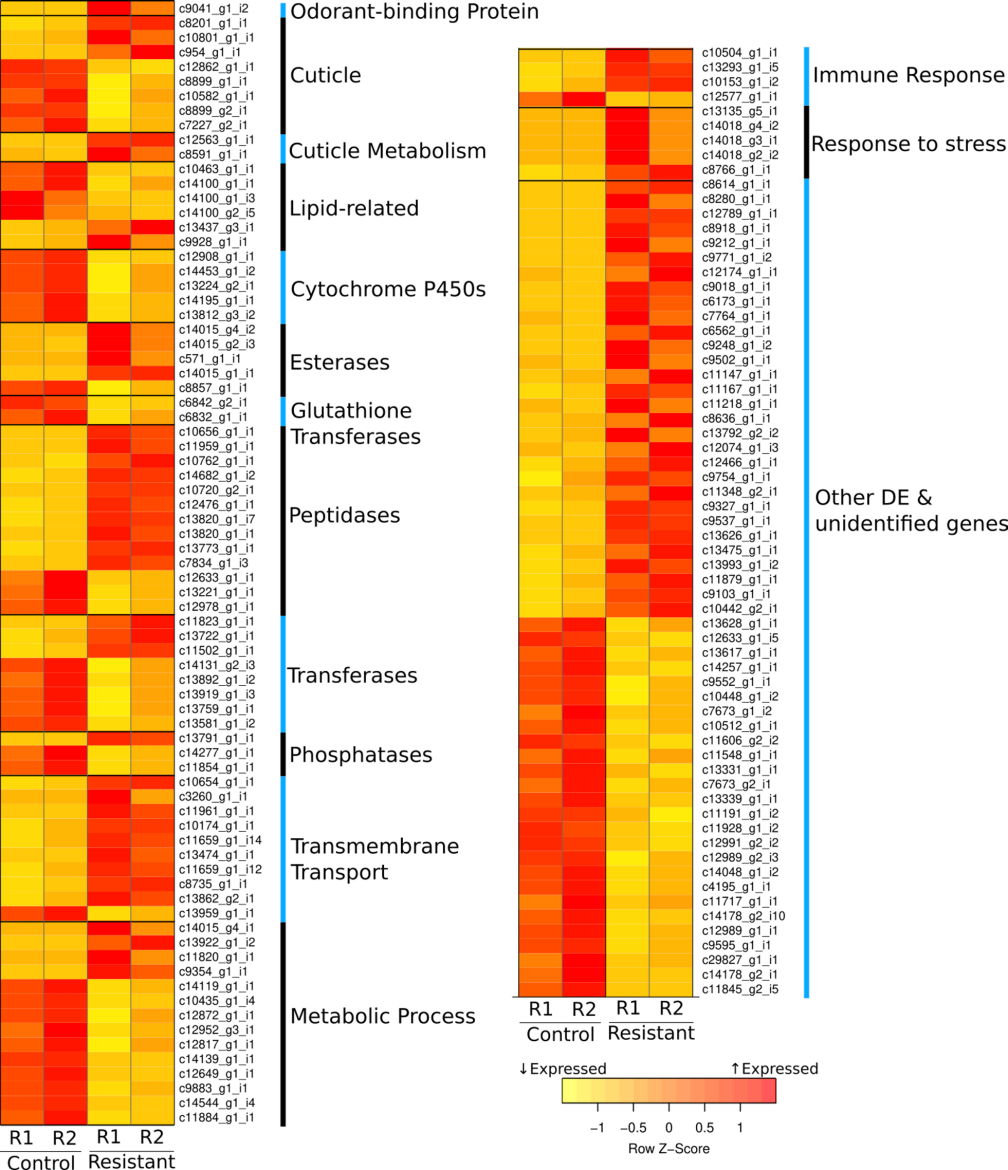
Heatmap of the transcripts (142) found differentially expressed between the control and resistant conditions, organized by functional categories. The expression abundances estimated by RSEM and eXpress were normalized by library size and averaged between both counting methods

Consistent with previous studies [44–51], heat-shock proteins (responsible for the enriched term “response to stress” in the Gene Ontology enrichment analysis) and immunity effectors were found over-expressed in the resistant condition (Fig. 2, Additional file 2: Dataset S1).

#### Metabolic responses

Also consistent with previous studies (see mini-review from [52]), esterase genes, known for their detoxifying role (reviewed in [53]), were found up-regulated in the resistant larvae (transcripts c14015_g1_i1, c14015_g2_i3, c14015_g4_i2, c571_g1_i1; Fig. 2 and Additional file 2: Dataset S1). Additionally, we found the GO terms “transmembrane transport”, “positive regulation of sodium ion transport”, “voltage-gated sodium channel complex”, “voltage-gated sodium channel activity”, and “extracellular region” (Fig. 2 and Additional file 3: Dataset S2) over-represented in the up-regulated transcript set. One possibility is that the up-regulation of trans-membrane transporters could help remove toxins from the cells and could therefore represent a non-specific response to the insecticide. More specific to *C. hominivorax*’s response to dimethyl-OP, genes involved in proteolysis (mostly serine-type endopeptidases) were found up-regulated in the resistant larvae (Fig. 2, Additional file 2: Dataset S1). Consistent with this result, the GO terms “proteolysis” and “serine-type endopeptidase activity” were found over-represented in the GO enrichment analysis (Additional file 3: Dataset S2). Finally, an odorant binding protein (c9041_g1_i2) playing a role in the sensory perception of chemical stimulus was found more than 8 times more expressed in the resistant condition compared to the control (Fig. 2 and Additional file 3: Dataset S2). It is possible that the insecticide binds specifically to this receptor.

In contrast with this result, we found a number of genes involved in metabolic processes, including putative glutathione transferases (c6832_g1_i1, c6842_g2_i1), a tryptophan phenylalanine hydroxylase (henna) (c14100_g2_i5, c14100_g1_i3, c14100_g1_i1) and cytochrome P450s (c14453_g1_i2, c12908_g1_i1, c13224_g2_i1, c14195_g1_i1, c13812_g3_i2), down-regulated in the resistant condition (Fig. 2 and Additional file 2: Dataset S1). This result was in agreement with the GO analysis, in which we found the GO terms “metabolic process”, “oxidation-reduction process”, “L-phenylalanine catabolic process”, “drug catabolic process” and “small molecule metabolic process” over-represented in the down-regulated transcript set (Additional file 3: Dataset S2).

#### Cuticle-related transcripts

Altered expression was observed for structural constituents of the cuticle and genes involved in the chitin metabolic process with some transcripts found up-regulated (c12563_g1_i1, c8591_g1_i1, c10801_g1_i1, c954_g1_i1, c8201_g1_i1), while others were down-regulated (c12862_g1_i1, c8899_g1_i1, c10582_g1_i1, c8899_g2_i1, c7227_g2_i1), suggestive of modification to the cuticle’s composition and properties (Fig. 2 and Additional file 2: Dataset S1).

### Variant analysis

Besides expression difference, it is possible that the resistance to dimethyl-OP insecticides is due to condition-specific polymorphisms (differential allelic expression or selected point mutations). Indeed, the original population (control condition) had individuals carrying the mutations Gly137Asp and/or Trp251Ser in the esterase E3 gene known to confer resistance. In particular, we expected to find homozygous individuals for the Trp251Ser mutation in the resistant group. This mutation was shown to increase the resistance specifically to dimethyl-OP [54], the insecticide used in this study.

**Fig. 3.**
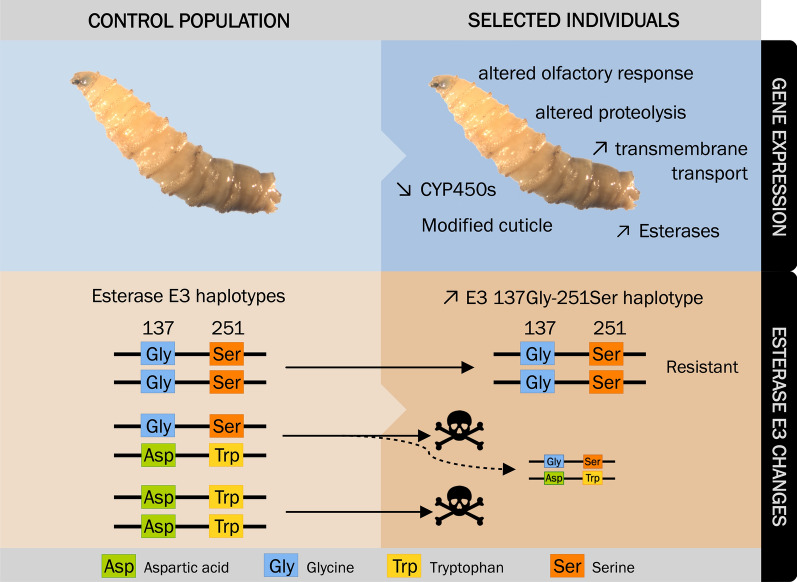
Diagram summarizing the molecular mechanisms associated with the resistance to dimethyl-OP insecticides. Main modulation of gene expression and differential allelic usage in resistant larvae (upper panel). Upward and downward arrows indicate up- and down-regulation in the resistant condition *versus* the control condition, respectively. Diagram of the Esterase E3 mutations at positions 137 and 251 in the control and resistant conditions (lower panel). Both mutations providing resistance to insecticides, 137Asp and 251Ser, are not found on the same haplotype. In the dimethyl-OP-resistant condition, we encountered almost invariably the 137Gly and 251Ser polymorphisms (see Table 1), which corroborate the findings of Carvalho et al. [25] that showed that 137Gly and 251Ser were on the same haplotype. It is to note that few 251Trp alleles were found in the resistant condition. We hypothesize that they come from heterozygous individuals (dashed line). The skull and bones represent the death of susceptible larvae exposed to the OP insecticide

As expected and in accordance with the Esterase E3 genotyping of Carvalho et al. [23], the resistant individuals had predominantly the esterase E3 (c13624_g1_i1) 251Ser allele (Table 1), which confers resistance to dimethyl-OPs [54]. We also detected a low frequency of the amino acid Leucine (Leu) at position 251 (251Leu allele) in resistant individuals (Table 1). The mutation Gly137Asp was found in the control condition (Table 1), however, the resistant individuals only presented the non-resistant 137Gly allele (Table 1). These observations led us to conclude that resistance-associated mutations at positions 137 and 251 are most likely not found on the same haplotype in this population (Fig. 3, lower panel). There was no evidence of recombination between these two sites in agreement with Carvalho *et al.* [1]. It is to note that we detected a few 251Trp alleles in the resistant condition (Table 1).

**Table 1:** Esterase E3 (transcript c13624_g1_i1) polymorphisms (positions 137 and 251) in the control and resistant conditions. Complete information on these transcripts and SNPs (including amino acid changes) can be found in Additional file 5: Dataset S4

	Codon (AA)	Control condition		Resistant condition	
		Rep. 1	Rep. 2	Rep. 1	Rep. 2
Position 137	GGG (Gly)	23 (43.4%)	404 (54.2%)	34 (81.0%)	482 (96.2%)
	GGC (Gly)	0 (0.0%)	0 (0.0%)	7 (16.6%)	19 (3.8%)
	GAC (Asp)	30 (56.6%)	341 (45.8%)	1 (2.4%)	0 (0.0%)
Position 251	TCG (Ser)	74 (48.7%)	503 (52.4%)	115 (95.8%)	558 (91.6%)
	TGG (Trp)	78 (51.3%)	444 (46.3%)	0 (0.0%)	34 (5.6%)
	TTG (Leu)	0 (0.0%)	0 (0.0%)	5 (4.2%)	17 (2.8%)
	TGT (Cys)	0 (0.0%)	12 (1.3%)	0 (0.0%)	0 (0.0%)

“AA” and “Rep.” stand for “Amino Acid” and “Replicate”, respectively. The amino acid three-letter abbreviations “Gly”, “Asp”, “Ser”, “Trp”, “Leu” and “Cys” stand for “Glycine”, “Aspartic acid”, “Serine”, “Tryptophan”, “Leucine” and “Cysteine”

With the present RNA-seq data, it was also possible to explore the expression of genes presenting condition-specific polymorphisms. Using the KisSplice [39] pipeline (See Methods), we identified 830 non-synonymous condition-specific polymorphisms within the coding region of 492 transcripts (1.5 SNPs/transcripts on average) (Additional file 4: Dataset S3). Of these transcripts, 256 (52%) displayed only one allele in the resistant sample. At present, it is unclear if in these cases, only one allele is expressed, or if individuals carrying the alternative allele died as a consequence of the insecticide treatment. Few transcripts (17) were found to be DE and have condition-specific SNPs (Table 2 and Additional file 5: Dataset S4). However, the GO enrichment analysis performed on the set of transcripts containing condition-specific SNPs revealed similar processes to the ones found in the DE GO analysis enrichment (Additional file 6: Dataset S5). In particular, we found several GO terms related to proteolysis/peptidase activity (“serine-type endopeptidase inhibitor activity”, “aminopeptidase activity”, “serine-type endopeptidase activity”, “negative regulation of endopeptidase activity”, “carboxypeptidase activity”) and metabolic processes (“chitin metabolic process”, “hormone metabolic process”, “cellular carbohydrate metabolic process”, “malate metabolic process”, “hexose metabolic process”) enriched in the transcripts with condition-specific SNPs. Also of interest, the GO terms “chitin binding” and “structural constituent of cuticle” were found over-represented. This result suggests that the processes related to the cuticle, metabolism and proteolysis are sensitive to dimethyl-OP insecticides in *C. hominivorax* and that some alleles are preferentially expressed depending on the condition or even necessary for the individual's survival when in contact with the insecticide.

**Table 2:** Annotated transcripts found DE and containing condition-specific polymorphisms

ID	log2FC	SNP positions	Common name (if available) and GO terms
c12633_g1_i1	−1.34	171, 212	GO:0007165 signal transduction, GO:0006508 proteolysis, GO:0006412 translation, GO:0051301 cell division GO:0005840 ribosome, GO:0003735 structural constituent of ribosome, GO:0004197 cysteine-type endopeptidase activity, GO:0000166 nucleotide binding
c12633_g1_i5	−1.31	171, 569, 212, 309, 294, 354	
c12908_g1_i1	−3.45	1362	cytochrome P450 - GO:0020037 heme binding, GO:0016705 oxidoreductase activity, acting on paired donors, with incorporation or reduction of molecular oxygen, GO:0004497 monooxygenase activity, GO:0005506 iron ion binding, GO:0055114 oxidation-reduction process
c13437_g3_i1	1.37	524	GO:0008289 lipid binding
c13628_g1_i1	−1.27	554	GO:0030246 carbohydrate binding
c13820_g1_i1	1.78	142, 343	GO:0004252 serine-type endopeptidase activity, GO:0010765 positive regulation of sodium ion transport, GO:0006508 proteolysis GO:0007586 digestion, GO:0005615 extracellular space, GO:0005886 plasma membrane
c13820_g1_i7	1.93	500	
c13993_g1_i2	1.35	185	GO:0008199 ferric iron binding, GO:0006879 cellular iron ion homeostasis, GO:0006826 iron ion transport, GO:0005576 extracellular region
c14119_g1_i1	−1.38	1006	GO:0004321 fatty-acyl-CoA synthase activity, GO:0004467 long-chain fatty acid-CoA ligase activity, GO:0016207 4-coumarate-CoA ligase activity , GO:0008756 o-succinylbenzoate-CoA ligase activity, GO:0009851 auxin biosynthetic process, GO:0009695 jasmonic acid biosynthetic process, GO:0001676 long-chain fatty acid metabolic process, GO:0005777 peroxisome
C14131_g2_i3	−1.33	1250	bifunctional 3'-phosphoadenosine 5'-phosphosulfate synthase - GO:0004781 sulfate adenylyltransferase (ATP) activity | GO:0005524 ATP binding | GO:0004020 adenylylsulfate kinase activity, GO:0016310 phosphorylation, GO:0000103 sulfate assimilation
c14544_g1_i4	−3.45	1177, 1597	GO:0004157 dihydropyrimidinase activity, GO:0051219 phosphoprotein binding, GO:0006212 uracil catabolic process, GO:0005737 cytoplasm
C8201_g1_i1	1.80	324	Cuticle 1 - GO:0042302 structural constituent of cuticle

Condition-specific polymorphisms were also found in transcripts potentially involved in olfactory behavior (c13758_g3_i4, c12483_g1_i2, c13091_g1_i2/4/9, c13818_g5_i3), pupation (c12606_g1_i1, c12483_g1_i2) and starvation (c12483_g1_i2, c12331_g1_i1), based on GO term assignment.

Of particular interest, the transcript c13758_g3_i4, corresponding to an odorant-binding protein (OBP), was found to contain five polymorphisms with frequency shifts between the resistant and control groups. This transcript, as well as the differentially expressed OBP c9041_g1_i2 mentioned earlier, could be involved in the perception of the insecticide.

For 227 transcripts, we found that the control condition predominantly expressed one allele (>90% of the gene expression was contributed from one allele) while the resistant condition expressed both. To validate these results, we generated 154,067,376 genomic paired sequences with an average length of 120 bp from the control population. Using these low coverage genomic reads, we could confirm that 105 of these transcripts (46,3%) were probably segregating in the original population, as both alleles were present in the DNA reads. In the RNA-seq data for these genes, one of the alleles is prevalent, while the genomic data from the same population shows both alleles. Hence, a differential allelic expression is the most plausible explanation, i.e. heterozygous individuals only expressed one of their two alleles. We hypothesize that the remaining transcripts (122) were also genetically heterozygous but had insufficient coverage to allow their detection in the genomic reads.

## Discussion

In this study, we compared the transcriptomes of second instar (L2) resistant and dimethyl-OP-treated larvae (resistant condition) to non-treated L2 larvae from a mixed population composed of susceptible and resistant individuals (control condition). The differential expression analysis revealed that only a few transcripts (142, 0.43%) had an altered expression between both conditions. In addition, we identified 492 transcripts with condition-specific polymorphisms. At this point, it is unclear if these polymorphisms are due to a differential allelic expression (part of the resistance would be due to phenotypic plasticity) or if individuals lacking certain polymorphisms died as a result of the OP-treatment (genotypic resistance). These transcripts were generally involved in similar processes, although only a few were found in both analyses.

From the differential allelic usage analysis, we could also confirm that the previously characterized Trp251Ser mutation in the esterase E3 gene [1, 21, 22] is necessary to confer resistance to dimethyl-OP insecticides (Fig. 3, lower panel), whereas the 137Asp mutation, that presumably confers resistance to dietyl-OP, was not present in the resistant individuals. These observations support the model that the 251Ser mutation is linked to the 137Gly wild-type polymorphism (Fig.3, lower panel). We hypothesize that the few transcripts with the wild-type 251Trp allele found in the resistant condition originated from 251Trp/251Ser heterozygous individuals (Fig. 3, lower panel). It is to note that we also found a few 251Leu alleles in resistant individuals, in agreement with Bergamo et al. [55], which showed this mutation in low frequencies in some populations of *C. hominivorax*. The Trp251Leu mutation was also identified in dimethyl-OP-resistant strains of *L. cuprina* [23] and could, therefore, have been present in the ancestor of *L. cuprina* and *C hominivorax*. The substitution of Tryptophan at position 251 by Serine or Leucine is specific for resistance to dimethyl-OPs, as demonstrated in *M. domestica* [54] and *L. cuprina* [56], respectively. Taşkin and colleagues [54] suggested that the substitution of Trp by smaller residues, such as Serine and Leucine, might enhance the hydrolysis of the dimethyl-OP by α-esterases, resulting in resistance. Although the esterase E3 carried the 251Ser mutation conferring resistance, no change in its expression was detected. However, four other esterases were found up-regulated in the resistant group (an exception to this was one thioesterase found down-regulated). We hypothesize that the up-regulation of these esterases (none of which appear to have polymorphisms) enhances the detoxification of the insecticide.

Other genes related to metabolic and detoxifying processes, including six cytochrome P450s (CYP450s), a family of genes known to be involved in resistance processes, were down-regulated in the larvae that survived the insecticide treatment. This result contrasts with many studies [57–60], which found that an over-expression of these genes conferred resistance to non-OP insecticides. However, these findings are in agreement with the findings of Carvalho *et al.* [22], which reported the under-expression of the Cyp6g1 Cytochrome P450 in OP resistant *C. hominivorax* individuals. Looking at differential allelic expression, we also found many transcripts involved in metabolic processes displaying condition-specific SNPs (7 CYP450s, 2 glutathione-S-transferases and numerous genes involved in proteolysis). Changes to the metabolism and detoxifying processes, especially the down-regulation of CYP450s can diminish the metabolization of the insecticide into its active form [61]. However, unlike the OP diazinon, which acts through its bioactive oxon metabolite, dichlorvos directly inhibits its target [62]. As such, dichlorvos does not require bioactivation and it is immediately toxic. Another possibility to explain our results is that the larvae display an aversion behavior towards insecticide-poisoned food, which could manifest itself by lower and/or altered metabolization rates. Down-regulation of detoxification genes, such as CYP450s, in the context of insecticide resistance is poorly understood. It was observed in mosquitoes resistant to permethrin [63] and could indicate an adaptive homeostatic response [64].

We also observed the up-regulation of heat-shock genes and immune effectors in the resistant condition. Heat-shock proteins (HSPs) allow organisms to adjust their tolerance levels and are produced by cells in response to exposure to environmental stresses. The up-regulation of HSPs are consistently reported in arthropods exposed to insecticides. It was observed in mosquitoes [44, 47, 51], flower thrips [49], the diamondback moth [48], and the Asian corn borer [45]. In agreement with previous studies [44, 46, 50], we observed a pattern of up-regulation of genes involved in the immune response after exposure to pesticides.

The altered expression and condition-specific polymorphisms of cuticle genes and genes involved in the chitin metabolic process is suggestive of a reduced penetration of the insecticide in the haemolymph by a thickening or a modification of the permeability of the cuticle, which is a common insecticide resistance mechanism (reviewed by [65]). Reduction of cuticular insecticide penetration is involved in the resistance of strains of the german cockroach, *Blattella germanica* [66, 67] and in the malaria vector, *Anopheles gambiae* [68]. Further experiments are needed to confirm the cuticle changes in *C. hominivorax* and their consequence on insecticide penetration.

Interestingly, we found an odorant binding protein (OBP) displaying condition-specific polymorphisms and another one was found highly up-regulated in the resistant larvae compared to the control ones. This result was concordant with the identification of condition-specific SNPs in genes putatively involved in olfactory behavior and starvation. In *Drosophila melanogaster*, OBPs modulate the ingestion of bitter tastants and can contribute directly to taste perception [69]. Lines expressing RNAi corresponding to putative orthologs of the OBP genes identified here, display altered feeding responses. Silencing of these genes in adult flies resulted in a reduction in feeding from sources with specific tastants [69]. Nucleotide changes in OBP sequences can also cause variation in olfactory behavior in response to chemical stimulus in *D. melanogaster* [70]. Changes in the expression of olfactory genes, such as the odorant-binding protein genes identified in this study, may indicate that resistant larvae are able to perceive the insecticide, which could trigger physiological responses and behavior avoidance of the toxic food. The behavioral avoidance, in turn, could lead to a reduced intake of the insecticide-contaminated food (starvation). Aversion to specific diets has been observed in screwworm larvae: they stop feeding, and this directly impacts the expression of genes involved in metabolic processes (unpublished results). Behavioral avoidance has been reported on multiple occasions and may be a pervasive resistance strategy in many cases [71, 72]. Resistant strains of *B. germanica*, for instance, avoid harborages treated with cypermethrin, while a susceptible strain apparently is unable to distinguish between treated and untreated harborages [73]. Resistant strains absorb a sublethal amount sufficient for detection, leading to successful avoidance of treated harborages, as they are not overwhelmed by the toxic effects of an insecticide. However, the molecular basis of behavioral resistance remains enigmatic and poorly characterized [72]. Here, we identified clear candidates for behavioral resistance (genes linked to olfactory, starvation and locomotion processes). Avoidance assays and food intake measurements using control and resistant larvae would have to be performed to test the magnitude of the behavioral resistance. If behavior resistance is significant, the specific OBP identified in this study could be targeted using the genetic engineering technique CRISPR/Cas9, which has been successfully carried out in *C. hominivorax* [74] or by using RNAi.

## Conclusions

In conclusion, it is clear that the Trp251Ser mutation in the esterase E3 is necessary to confer resistance to dimethyl-OP insecticides, however it is uncertain if it is sufficient to provide full dimethyl-OP resistance. The up-regulation of other esterases, as well as the avoidance of insecticide intake, may be important complementary strategies and ought to be considered in our current pest management strategies. Finally, our study demonstrates that the resistance and response mechanisms to dimethyl-OP insecticides involve not only modulations of the expression of genes but also the presence of condition-specific polymorphisms, which is a generally overlooked mechanism in RNA-seq analyses.

## Supplementary information

**Additional file 1: Table S1.** Number of reads in the different samples before and after trimming and collapsing.

**Additional file 2: Dataset S1.** Differentially Expressed (DE) transcripts between the control and resistant conditions and their annotations. DE transcripts were those displaying a log2 of the Fold Change (FC) greater than 1 and an adjusted P-value (FDR) lower than 0.05 in all analyses performed (combinations between the counting methods eXpress or RSEM and the testing packages EdgeR and DEseq2). In blue and red are the transcripts found down- and up-regulated in the resistant condition versus the control condition. Negative and positive log2FC indicate whether the transcripts were found down- or up-regulated in the resistant condition versus the control one.

**Additional file 3: Dataset S2.** Gene Ontology (GO) terms found over-represented in the transcripts found differentially expressed using the enrichment analysis implemented in BLAST2GO. Sheet 1 and sheet 2 correspond to the complete and reduced sets of GO terms found enriched in the DE transcripts sets, respectively. The reduced list derives from the reduction of the complete list of enriched GO terms to its most specific terms, using BLAST2GO’s functionality. ‘Nr Test’ and ‘Nr Reference’ refer to the number of transcripts annotated with the corresponding term in the test set and the Reference set, respectively. ‘Non Annot’ and ‘Non Annot Reference’ refer to the number of transcripts not annotated with the corresponding GO terms in the test set and reference set, respectively.

**Additional file 4: Dataset S3.** Condition-specific polymorphisms between the resistant and control conditions with transcript annotation information. The KisSplice pipeline (see Methods) was used to identify condition-specific polymorphisms (SNPs). We only considered SNPs displaying an adjusted P-value below 0.01, a minimum coverage of 20 per replicate and condition and a magnitude of the differential allelic usage (Absolute Deltaf/DeltaPSI) above 0.2 (20%). Only polymorphisms within the coding region of the transcript and found non-synonymous were further analyzed. Columns 3 to 10 are the normalized read counts of each variant for each replicate and condition. Annotation was performed using FunctionAnnotator (see Methods).

**Additional file 5: Dataset S4.** Transcripts found differentially expressed (DE) and displaying condition-specific polymorphisms with their annotations. Annotations were obtained using FunctionAnnotator. For gene expression abundances, we used Express and RSEM. For differential expression testing and differential allelic usage, we used EdgeR, DEseq2 and KisSplice (See Methods).

**Additional file 6: Dataset S5.** Gene Ontology (GO) terms found over-represented in the transcripts containing condition-specific SNPs. Sheet 1 and sheet 2 correspond to the complete and reduced sets of GO terms found enriched in the DE transcripts sets, respectively. The reduced list derives from the reduction of the complete list of enriched GO terms to its most specific terms, using BLAST2GO’s functionality. ‘Nr Test’ and ‘Nr Reference’ refer to the number of transcripts annotated with the corresponding term in the test set and the Reference set, respectively. ‘Non Annot’ and ‘Non Annot Reference’ refer to the number of transcripts not annotated with the corresponding GO terms in the test set and reference set, respectively.

## Data Availability

The raw sequencing data as well as transcript abundance measurements were deposited in the GEO (Gene Expression Omnibus) repository under the accession number GSE145822.

## Abbreviations

AChE: Acetylcholinesterase
Asp: Aspartic acid
cDNA: Complementary DNA
CRISPR/Cas9: Clustered Regularly Interspaced Short Palindromic Repeats associated protein-9 nuclease
CYP450s: Cytochrome P450s
DDT: Dichlorodiphenyltrichloroethane
DE: Differential expression
OP: Organophosphate (OP)-based insecticides
Dimethyl-OP: 2,2-Dichlorovinyl dimethyl phosphate C4H7Cl2O4P
FDR: False discovery rate
Gly: Glycine
GO: Gene Ontology
LC90: Lethal concentration for 90% of the population
Leu: Leucine
SNPs: Single nucleotide polymorphism
qPCR: Quantitative polymerase chain reaction
RNA-seq: RNA-sequencing
Ser: Serine
Trp: Tryptophan
