# Supplementary material for: Molecular basis of resistance to organophosphate insecticides in the New World screw-worm fly
Source: Parasit Vectors. 2020 Nov 10;13:562. doi: 10.1186/s13071-020-04433-3 (PMC7653728; doi:10.1186/s13071-020-04433-3)
Supplement: Supplementary file 1 — Additional file 1: Table S1. Number of reads in the different samples before and after trimming and collapsing. [file 13071_2020_4433_MOESM1_ESM.docx]

**Additional file 1: Supplementary table S1. Number of reads in the different samples before and after trimming and collapsing**

| **Assay** | **Condition** | **Raw reads** | **After trimming**  **(% kept)** | **Collapsed (% of trimmed reads kept)** |
| --- | --- | --- | --- | --- |
| Replicate 1  (Single-end) | Control | 15,427,065 | 11055926 (84.86%) | 4,806,021 (43.47%) |
|  | Resistant | 17,021,595 | 11279633 (84.64%) | 5,845,907 (51.83%) |
| Replicate 2  (Paired-end) | Control | 26,807,116 | 26277392 (98.02%) | 20,453,006 (77.83%) |
|  | Resistant | 24,558,107 | 24091747 (98.10%) | 19,130,494 (79.41%) |
